# The impact of COVID-19 on myocardial infarctions, strokes and out-of-hospital cardiopulmonary arrests: an observational retrospective study on time-sensitive disorders in the Friuli Venezia Giulia region (Italy)

**DOI:** 10.1186/s12245-022-00473-x

**Published:** 2022-12-29

**Authors:** Carlo Pegani, Giovanni Buttignon, Annarita Tullio, Marcello Naccarato, Paolo Manganotti, Serena Rakar, Enrico Fabris, Federico Nadalin, Vincenzo Mione, Gian Luigi Gigli, Simone Lorenzut, Leonardo Spedicato, Paolo Passadore, Daniela Pavan, Cristina Lutman, Manila Andrian, Massimo Borelli, Stefano Novello, Rita Belfiore, Chiara Daneluzzi, Gianfranco Sinagra, Alberto Peratoner

**Affiliations:** 1 Azienda Sanitaria Universitaria Giuliano Isontina, Trieste, Italy; 2 Azienda Sanitaria Universitaria Friuli Centrale, Udine, Italy; 3 Azienda Regionale di Coordinamento della Salute, Udine, Italy; 4 Azienda Sanitaria Friuli Occidentale, Pordenone, Italy; 5 UMG School of PhD Programmes, University Magna Graecia of Catanzaro, Catanzaro, Italy

**Keywords:** COVID-19, Mortality, Acute myocardial infarction, Stroke, Cardiopulmonary arrest, Emergency medical service (EMS), Cardiopulmonary resuscitation

## Abstract

The COVID-19 global pandemic has changed considerably the way time-sensitive disorders are treated. Home isolation, people’s fear of contracting the virus and hospital reorganisation have led to a significant decrease in contacts between citizens and the healthcare system, with an expected decrease in calls to the Emergency Medical Services (EMS) of the Friuli-Venezia Giulia (FVG) region. However, mortality in clinical emergencies like acute ST-elevation myocardial infarction (STEMI), stroke and out-of-hospital cardiopulmonary arrest (OHCA) remained high. An observational retrospective cross-sectional study was carried out in FVG, taking into account the period between March 1, 2020, and May 31, 2020, the first wave of the COVID-19 pandemic, and comparing it with the same period in 2019. The flow of calls to the EMS was analysed and COVID-19 impact on time-sensitive disorders (STEMIs, ischemic strokes and OHCPAs) was measured in terms of hospitalisation, treatment and mortality. Despite a −8.01% decrease (*p* value ˂0.001) in emergency response, a 10.89% increase in calls to the EMS was observed. A lower number of advanced cardiopulmonary resuscitations (CPR) (75.8 vs 45.2%, *p*=0.000021 in April) and ROSC (39.1 vs 11.6%, *p*=0.0001 in April) was remarked, and survival rate dropped from 8.5 to 5%. There were less strokes (−27.5%, *p* value=0.002) despite a more severe onset of symptoms at hospitalisation with NHISS˃10 in 38.47% of cases. Acute myocardial infarctions decreased as well (−20%, *p* value=0.05), but statistical significances were not determined in the variables considered and in mortality. Despite a lower number of emergency responses, the number of calls to the EMS was considerably higher. The number of cardiac arrests treated with advanced CPR (ALS) was lower, but mortality was higher. The number of strokes decreased as well, but at the time of hospitalisation the clinical picture of the patient was more severe, thus affecting the outcome when the patient was discharged. Finally, STEMI patients decreased; however, no critical issues were observed in the variables taken into account, neither in terms of response times nor in terms of treatment times.

## Introduction

In the autumn of 2019, the first COVID-19 case in Wuhan (China) marked the outbreak of the pandemic and in a short amount of time the world’s population would find itself in an emergency state. In Italy, the lockdown came into force on March 9, 2020, and citizens were asked to stay home in order to avoid contracting the virus and spreading it.

Hospitals could not help but adapt to the new infectious disease by closing some wards, turning some into COVID-19 wards and abolishing non-urgent surgeries. The regional EMS, which are the first point of contact with the residents, were hugely affected in their activities, which meant a lower number of emergency responses despite having the same, or even higher number of calls [1].

Previous studies carried out in the USA [2], referring to the same period of time of this study, showed on one side a significant decrease in the daily number of emergency calls for cardiovascular [3, 4] (−1.2%) and neurological emergencies (−12.3%), as just demonstrated in another previous study [5] and on the other side an increase in calls for cardiac arrests (+18.2%) and a lower number of ROSC cases (−16.2%), without observing a remarkable difference regarding age and sex of the patients.

Measuring these aspects is crucial not only to compare available data in literature with those collected by the scientists in this study, but also to describe and perhaps explain the phenomenon. The main aim of this study was to compare mortality rate in STEMI, stroke and OHCA patients aged 18 and over and treated by EMS during the two times intervals taken into account.

The study was also aimed at comparing the number of STEMI, stroke and OHCPA cases during and before the pandemic wave, analysing a potential decrease in the number of cardiac arrests and ROSC, describing the neurological picture of a patient affected by a stroke, by indicating the number of times that the Stroke Protocol was activated and was followed by a thrombolytic therapy, and finally describing the number of STEMI patients who reached out to EMS and response times.

## Materials and methods

An observational retrospective cross-sectional study was carried out. The study takes into account STEMI, STROKE and OHCA patients aged 18 and over and treated by EMS during the first wave of the COVID-19 pandemic, from March 1, 2020, to May 31, 2020, and during the same period of 2019 (pre-COVID). The following patients were excluded: patients aged under 18, OHCA victims who have not been resuscitated, patients found dead, those with expected deaths (DNR orders, terminal illness or other serial medical conditions) and those to whom thrombolytic therapy was not administered.

Data were collected by scientists in different Excel database. The researchers of the four agencies that took part in the cross-sectional study collected data from the pathology registries of each unit retrospectively in 2021. The collection process was followed by the creation of a single document that contained all the information.

The study was conducted in accordance with the GCP, the ethical principles deriving from the declaration of Helsinki and the current legislation on observational studies. The IRB code of the study is CEUR-2022-OS-10.

### Setting

Data belonging to the FVG healthcare agencies were collected and investigated. The following agencies took part in the study: the *Azienda Sanitaria Universitaria Giuliano Isontina* (ASUGI), the *Azienda Sanitaria Universitaria Friuli Centrale* (ASUFC), the *Azienda Sanitaria Friuli Occidentale* (ASFO) and the *Azienda Regionale di Coordinamento della Salute* (ARCS). More specifically, this study saw the participation of the haemodynamic laboratories, the stroke units of Trieste, Udine and Pordenone, the regional Emergency Operations Centre (SORES), which handles FVG emergency calls, and the *Struttura Semplice Dipartimentale Pronto Soccorso Ospedale Maggiore e Gestione delle Urgenze Territoriali di Trieste* (in short it is the ED in charge of the Trieste area).

### Data analysis

Data analysis was performed using the R and JASP software. Statistical description of outcomes and covariates was carried out by means of absolute and relative frequencies with regard to nominal and categorical variables; with regard to continuous variables or variables associated to different levels of measure, we used mean and standard or median deviations, interval and interquartile ranges depending on the distribution shape, which was assessed through Kolmogorov-Smirnov test. Incidence rate and relations among incidence rates with a 95% relative confidence interval (CI 95%) were calculated.

In order to investigate the differences between the two above-reported periods of time and among the four provinces (Udine, Trieste, Gorizia and Pordenone), chi-square test and Fisher’s exact test were conducted based on the number in the cell with regard to categorical and qualitative variables, whereas for independent samples, *t* test or Wilcoxon-Mann Whitney test were used, based on the distribution frequency of data with regard to quantitative variables related to dichotomous variables. One-way ANOVA or Kruskal-Wallis tests were used in terms of distribution frequency of data, with regard to quantitative variables related to categorical variables with more than two levels. Test *z* was used to make a comparison in terms of proportions. *P* value of two-tailed test <0.05 was considered to be statistically significant.

## Results

Comparing March, April and May 2019 and 2020, with regard to all the calls handled by SORES, a considerable increase in the number of calls during the pandemic period compared to the pre-pandemic period has to be seen: 77740 calls to SORES in 2020 vs 70103 (+10.89%, *p* value <0.001–*z* test) in 2019. More specifically, an increased number of emergency calls was registered in March 2020 (+31.69%).

Despite a 10.89% increase in calls, the number of emergency responses decreased by 8.7% during the lockdown (27,817 vs 30,240; −2423, −8.01%, *p* value <0.001 – *z* test).

By analysing the flow of emergency calls, differentiated into the above-reported disorders, it can be said that as far as chest pain symptoms are concerned, an important increase in calls during the COVID-19 pandemic was observed: 2279 vs 2012 during the pre-pandemic period (+6.2%, *p* value <0.001–*z* test). A turnaround was noticed regarding strokes, with a decrease in calls in 2020 compared to 2019: 812 cases in 2020 compared to 860 cases in 2019 (−5.5%). With regard to the total number of OHCAs, with SORES nurses giving pre-arrival instructions, no difference in the incidence rate was registered in the period taken into account (297 events in both quarters).

By comparing the total number of OHCAs with those where advanced CPR was performed, a significant statistical difference emerged in March (chi-square test, *p*=0.009422), in April (chi-square test, *p*=0.00002) and in May (chi-square test, *p*=0.004393) (Table 1).

**Table 1:** OHCAs with ALS/TTOTAL OHCAs (n°/%)

Year	Month					
	March	%	April	%	May	%
2019	57/104	54.8	69/91	75.8	62/102	60.7
2020	40/108	37	43/95	45.2	38/94	40.4

However, the analysis of the event distribution, with regard to the provinces where it took place, allowed a +17.75% increase in the province of Udine, a −5% and a −13.6% decrease in OHCAs in the Trieste and Pordenone provinces, respectively, to be registered. In the province of Gorizia, no considerable difference was observed during the two times frames (+0.2%) (Table 2).

**Table 2:** 2019 vs. 2020, OHCAs with ALS/province

Province	March			April			May		
	2019	2020	***p***	2019	2020	***P***	2019	2020	***p***
Udine	24	23	0.88	29	27	0.79	24	21	0.66
Trieste	12	4	0.045	14	9	0.3	13	6	0.11
Gorizia	6	7	0.78	11	2	0.0126	6	6	1
Pordenone	15	6	0.049	15	5	0.025	19	5	0.004

By analysing ROSC variable, it emerged that in 2020, 18 ROSC were achieved out of 121 CPRs (14.9%) compared to the 2019 quarter when ROSC was achieved in 51 cases out of 188 CPRs (27.1%). By furtherly dividing ROSC variable into the above-reported months, we can see that the difference is more significant in April (chi-square test, *p*=0.00001) than in March (chi-square *p*=0.089) and May (chi-square test, *p*=0.38) (Table 3).

**Table 3:** ROSCs/OHCAs with ALS (n°%)

Year	Month					
	March	%	April	%	May	%
2019	14/57	24.5	27/69	39.1	10/62	16.1
2020	7/40	17.5	5/43	11.6	6/38	15.7

Finally, the analysis of survival rate showed a directly proportional relationship between survival rate and the variables taken into account: during the 1st quarter of the COVID-19 pandemic, the survival rate was 5% compared to 8.5% registered during the same time frame in 2019. Table 4 (event type) sums up STEMI and stroke cases that have taken place in the 2019–2020 time frame: there is a significant similarity (chi-square test, *p*=0.05151) between the total number of events on annual basis according to the type (neurological vs cardiac). More specifically, with regard to the neurological events observed in the period taken into account on regional-scale, 233 patients were admitted during the pre-pandemic period compared with 169 patients in 2020 (−27.5%), which signified a considerable reduction in stroke events (*z* test, *p* value=0.0014).

**Table 4:** Event type

Year	Type	Frequency	%
2019	STEMI	171	42.3
	STROKE	233	57.7
	Total	404	100
2020	STEMI	137	44.8
	STROKE	169	55.2
	Total	306	100

No significant difference in terms of patients’ age was observed (2019=±76 vs 2020=±75). Instead, a reduction in Stroke Protocol activations was noticed (233 vs 169) and an unvaried use of thrombolytic therapy was detected during the pandemic (60, 35.50%) compared with the pre-pandemic period (83, 35.17%). A higher frequency of severe neurological events at the time of admission (NIHSS>10) was remarked in 2020 (65, 38.47%) in comparison with 2019 (81, 34.32%), this negatively influenced the outcomes.

With regard to both the total number of events and STEMI and stroke events (*z* test, *p*=60 and 0.82, respectively) and by analysing data according to the provinces, no significant variation was remarked in the Pordenone province (*z* test, *p*=0.59). Instead, as far as Trieste and Udine are concerned, a significant variation emerged with regard to the total number of events (*z* test, *p*=0.02 and 0.001, respectively). The same could not be said about STEMI and stroke events (in Udine a significant variation was observed in terms of stroke, *z* test *p*=0.002) (Table 5).

**Table 5:** Distribution by province

		Year			*p* value two-proportions *z* test
City	Type	2019	2020	Total	
Pordenone	STEMI	47	42	89	**0.60**
	STROKE	41	39	80	**0.82**
	Total	88	81	169	**0.59**
Trieste	STEMI	59	43	102	**0.11**
	STROKE	62	45	107	**0.10**
	Total	121	88	209	**0.02**
Udine	STEMI	65	52	117	**0.23**
	STROKE	130	85	215	**0.002**
	Total	195	137	332	**0.001**
Total	STEMI	171	137	308	**0.05**
	STROKE	233	169	402	**0.001**
	Total	404	306	710	**0.0002**

While considering cardiac events, during the time frame taken into account, it emerged that 137 events were registered during the COVID-19 pandemic compared with 171 events during the same period of time in 2019 (−20%, *p*=0.05). By analysing each subgroup, STEMI admissions dropped in all three hubs of the Friuli-Venezia Giulia region. More specifically, the following reductions were detected: 10.6% in the Pordenone province, 27% in the Trieste province and 20% in the Udine province. Nevertheless, no remarkable statistical difference emerged (*p* value di 0.6 for Pordenone, 0.11 for Trieste, 0.23 for Udine, 0.05 by taking into account the total number, two-proportion *z* test; *p* value 0.78 chi-square test by taking into account the province and the year). No difference was detected in terms of patients’ average age (65.5 ± 12.7 in 2019 vs 67.8 ±12.4 in 2020, *p* value=0.09, Wilcoxon-Mann Whitney test). Hospitalisations drop did not refer to a specific sex: in both women and men, a proportional reduction was remarked (*p* value=0.69–chi-square test).

During the COVID-19 period, with regard to potential delays in alerting the emergency services, the analysis of timing during STEMIs brought to light variations with no statistically significant difference neither in terms of timing from onset of symptoms to coronary angiography (*p* value=0.07, Wilcoxon-Mann Whitney test) nor in terms of timing from pain manifestation to the emergency call (*p* value=0.095, Wilcoxon-Mann Whitney test).

No significant results emerged from the other considered variables. More specifically, no variations were seen with regard to the number of OHCA, which were attended in the haemodynamic laboratories (*p* value=0.72, chi-square test), with regard to the number of cardiogenic shocks (*p* value=0.33 chi-square test) and finally with regard to the number of patients for whom endotracheal intubation was needed (*p* value=0.56 chi-square test).

After discharging the patient, STEMI mortality remained stable, with an 8.2% mortality rate in 2019 compared with 9.5% in 2020 (*p* value=0.6881, chi-square test).

Instead, with regard to mortality of STEMI or stroke patients on an annual basis, no statistically remarkable difference was noticed (Table 6).

**Table 6:** Mortality

		Year					***p*** value
City	Type	2019		2020		Total	Chi-square test
		Survival	Death	Survival	Death		
**Pordenone**	STEMI	40	7	36	6	89	**0.94**
	STROKE	38	3	33	6	80	**0.31**
**Trieste**	STEMI	55	4	40	3	102	**1**
	STROKE	50	12	41	4	107	**0.17**
**Udine**	STEMI	62	3	48	4	117	**0.7**
	STROKE	124	6	81	4	215	**1**
**Total**	STEMI	157	14	124	13	308	**0.69**
	STROKE	212	21	155	14	402	**0.8**
**Total**		369	35	279	27	710	**0.94**

## Discussion

Home isolation necessary to contain the spread of COVID-19 fundamentally disrupted people’s lives. The COVID-19 pandemic wave has profoundly changed the contacts between citizens and the healthcare system not only because of citizens’ fear of contracting the virus but also because of hospital reorganisation (reorganisation of hospital operations, former COVID-free wards being turned into COVID-19 wards with a reduction in inpatient care beds) [1].

Furthermore, the psychological aspect concerning the health personnel involved in out-of-hospital emergencies must not be forgotten. Never as in this period has represented the joining link between the citizens and the hospital. EMS personnel have undergone an additional load of stress associated with the fear of contracting the virus, the attention in the correct dressing with personal protective equipment, the need to carry out advanced maneuvres in extremely difficult conditions. This aspect also emerges from the literature in which COVID-19 had a profound impact on the time-dependent OHCA network where during 2019–2020 there was a significative reduction also in CPRs performed by bystanders [6, 7].

As a results from the analyses, a higher number of emergencies calls with a lower number of cardiac arrests treated with advanced CPR (this has been showed literature [8, 9]) and a lower number of strokes and STEMIs in the whole Friuli-Venezia Giulia region during the COVID-19 pandemic was observed.

An increased flow of calls to SORES was not associated to a higher number of emergency responses, which on the contrary dropped, as confirmed by the literature [10]. This can be explained by the fact that many people called SORES not because they had predictive symptoms of cardiac arrest, stroke and STEMI, compared to the pre pandemic period [11], but in order to receive information in case they tested positive for COVID.

This aspect can be associated to the spread of information by media according to which hospitals were the places with the highest possibility of contracting the virus because of the lack of Personal Protective Equipment (PPE) among healthcare workers.

With regard to cardiac arrests, despite a total number that has not changed during the two times frames taken into account, variations were detected in the number of cardiac arrests treated with ALS, in OHCA distribution according to the province and in the number of ROSC. In the Friuli-Venezia Giulia region in its whole, these data decreased, and unfortunately so did patients’ survival rate.

Neurological results in stroke patients, regardless of having or not received thrombolytic therapy (criteria for Stroke Protocol activation are shared on regional scale in a document with clinical attendance instructions), worsened during lockdown, which suggests that, despite a decreased flow of calls for this disorder in the 2020 quarter, a probable delay in calling healthcare professionals caused worst clinical situations compared to those observed during the pre-COVID period [12, 13].

From a cardiac point of view as well, a general decrease in STEMI admissions was registered in FVG during the COVID-19 pandemic. Nevertheless, by analysing the variables that take into account, the timing from onset of symptoms to coronary procedure and timing from the first emergency call to revascularisation, no specific variations emerged. This result turns out to be in contrast with the results emerged from other studies [14, 15], in which an increase in both timings was noticed. This data shows how protocols to access the healthcare departments did not slow down patient’s access to the stroke units or to the haemodynamic laboratories.

Moreover, with regard to hospital reorganisation, it must be underlined that the number of beds for stroke or for STEMI patients was never reduced. Consequently, a decreased number in the access to stroke units or to haemodynamic laboratories have nothing to do with this aspect. Furthermore, it cannot be excluded that a decreased number of events concerning all three disorders taken into account was partially due to a lower physical stress and a higher rest imposed by lockdown. With regard to OHCAs and strokes, a potential delay in alerting the EMS has negatively influenced both the outcomes and the survival rate [16, 17].

In fact, other searches demonstrate lower outcomes on time-sensitive disorders with a higher time to contact to EMS [18], but whit a stable trend for visits and hospitalisation in non-COVID perido compared with this one [19].

An increased mortality was observed with regard to cardiac arrests, with 5% survival rate. Instead, stroke and STEMI patients’ mortality was not subject to any considerable variations, showing how EMS withstood the impact of the reorganisation of the healthcare agencies, by constantly assuring high levels of care and by preventing avoidable deaths.

## Conclusions

Despite a lower number of emergency responses, the number of calls to the EMS was considerably higher. The number of cardiac arrests treated with advanced CPR (ALS) was lower, but mortality was higher. The number of strokes decreased as well, but at the time of hospitalisation, the clinical picture of the patient was more severe, thus affecting the outcome when the patient was discharged. Finally, STEMI patients decreased; however, no critical issues were observed in the variables taken into account, neither in terms of response times nor in terms of treatment times.

### Limitations

The study is subject to some limitations. It is an observational retrospective study whose data were first collected by many scientists in different operational facilities and then gathered, which affected the timing this article was drawn up. It is important to underline that the pandemic, which has lasted for 2 years, challenged the process of data collection, which clearly affected variables taken into account. In spite of this, it was still decided to analyse the quarter of the first COVID-19 wave. Finally, as this is an observational study, determining cause-and-effect relationships is not possible.

## Data Availability

The datasets generated during and/or analysed during the current study are available from the corresponding author on reasonable request.

## References

[1] Munjal KG, Silverman RA, Freese J (2011). Utilization of emergency medical services in a large urban area: description of call types and temporal trends. Prehosp Emerg Care.

[2] Goldeberg Scott A, Cash RE, Peters G, et al. The impact of COVID-19 on statewide EMS use for cardiac emergencies and stroke in Massachusetts, December 1,2020. J Am Coll Emerg Physicians Open. 2021;2:e12351. 10.1002/emp2.12351 PMC7823089 33532755

[3] Roffi M, Guagliumi G, Ibanez B (2020). The obstacle course of reperfusion for ST-segment-elevation myocardial infarction in the COVID-19 pandemic. Circulation..

[4] Baracchini C, Pieroni A, Viaro F (2020). Acute stroke management pathway during coronavirus-19 pandemic. Neurol Sci.

[5] Wong L, Hawkins J, Langness S, Murrell K, Iris P, Sammann A (2020). Where are all the patients? Addressing COVID-19 fear to encourage sick patients to seek emergency care. NEJM Catalyst.

[6] Stirparo G, Fagoni N, Bellini L, at all. (2022). Cardiopulmonary resuscitation missed by bystanders: collateral damage of coronavirus disease 2019. Acta Anaesthesiol Scand.

[7] Nishiyama C, Kiyohara K, Kitamura T (2022). at all, Impact of the COVID-19 pandemic on prehospital intervention and survival of patients with out-of-hospital cardiac arrest in Osaka City, Japan. Circ J.

[8] Hasani-Sharamin P, Saberian P, Sadeghi M, et al. Characteristics of emergency medical service missions in out of hospital cardiac arrest and death cases in the periods of before and after the COVID-19 pandemic. Iran: Cambridge University Press; 2021. 10.1017/S1049023X21001138 PMC8529353 34622749

[9] Marijon E, Karam N, Jost D (2020). Out-of-hospital cardiac arrest during the COVID-19 pandemic in Paris, France: a population based, observational study. The Lancet. Public Health.

[10] Fussman C, Rafferty AP, Lyon-Callo S, Morgenstern LB, Reeves MJ (2010). Lack of association between stroke symptom knowledge and intent to call 911: a population-based survey. Stroke..

[11] Naccarato M, Scalia I, Olivo S, et al. Has COVID-19 played an unexpected “stroke” on the chain of survival? Italy: Elsevier B.V; 2020. 10.1016/j.jns.2020.116889 PMC7201240 32416370

[12] Wang Y, Shu H, Liu H (2021). The peak levels of highly sensitive troponin I predicts in-hospital mortality in COVID-19 patients with cardiac injury: a retrospective study. Eur Heart J Acute Cardiovasc Care.

[13] Cosentino N, Assanelli E, Merlino L, Mazza M, Bartorelli AL, Marenzi G. An inhospital pathway for acute coronary syndrome patients during the COVID-19 outbreak: initial experience under real-world suboptimal conditions. Can J Cardiol. 2020. 10.1016/j.cjca.2020.04.011. 10.1016/j.cjca.2020.04.011 PMC7162765 32376346

[14] De Rosa S, Spaccarotella C, Basso C, et al. Reduction of hospitalizations for myocardial infarction in Italy in the COVID-19 era. Italy: ESC, European Society of Cardiology; 2020. 10.1093/eurheartj/ehaa409 PMC7239145 32412631

[15] Stefanini GG, Alaide C (2020). ST-Elevation myocardial infarction in patients with COVID-19 clinical and angiographic outcomes. Circulation.

[16] Zou F, Qian Z, Wang Y, Zhao Y, Bai J (2020). Cardiac injury and COVID-19: a systematic review and meta-analysis. CJC Open.

[17] Zuin M, Rigatelli G, Zuliani G (2020). Incidence and mortality risk in coronavirus disease 2019 patients complicated by acute cardiac injury: systematic review and meta-analysis. J Cardiovasc Med (Hagerstown).

[18] Kim SH, Cruz SD, Conrardy JM, et al. Emergency department visits for serious diagnoses during the COVID-19 pandemic. USA: Wiley Public Health Emergency Collection; 2020. 10.1111/acem.14099 PMC7436489 32737912

[19] Santi L, Golinelli D, Tampieri A (2021). Non-COVID-19 patients in times of pandemic: emergency department visits, hospitalizations and cause-specific mortality in Northern Italy. PLoS One.

